# Methy-Pipe: An Integrated Bioinformatics Pipeline for Whole Genome Bisulfite Sequencing Data Analysis

**DOI:** 10.1371/journal.pone.0100360

**Published:** 2014-06-19

**Authors:** Peiyong Jiang, Kun Sun, Fiona M. F. Lun, Andy M. Guo, Huating Wang, K. C. Allen Chan, Rossa W. K. Chiu, Y. M. Dennis Lo, Hao Sun

**Affiliations:** 1 Centre for Research into Circulating Fetal Nucleic Acids, Li Ka Shing Institute of Health Sciences, The Chinese University of Hong Kong, Shatin, New Territories, Hong Kong SAR, China; 2 Department of Chemical Pathology, The Chinese University of Hong Kong, Prince of Wales Hospital, Shatin, New Territories, Hong Kong SAR, China; 3 Department of Obstetrics and Gynaecology, The Chinese University of Hong Kong, Prince of Wales Hospital, Shatin, New Territories, Hong Kong SAR, China; Wayne State University, United States of America

## Abstract

DNA methylation, one of the most important epigenetic modifications, plays a crucial role in various biological processes. The level of DNA methylation can be measured using whole-genome bisulfite sequencing at single base resolution. However, until now, there is a paucity of publicly available software for carrying out integrated methylation data analysis. In this study, we implemented Methy-Pipe, which not only fulfills the core data analysis requirements (e.g. sequence alignment, differential methylation analysis, *etc*.) but also provides useful tools for methylation data annotation and visualization. Specifically, it uses Burrow-Wheeler Transform (BWT) algorithm to directly align bisulfite sequencing reads to a reference genome and implements a novel sliding window based approach with statistical methods for the identification of differentially methylated regions (DMRs). The capability of processing data parallelly allows it to outperform a number of other bisulfite alignment software packages. To demonstrate its utility and performance, we applied it to both real and simulated bisulfite sequencing datasets. The results indicate that Methy-Pipe can accurately estimate methylation densities, identify DMRs and provide a variety of utility programs for downstream methylation data analysis. In summary, Methy-Pipe is a useful pipeline that can process whole genome bisulfite sequencing data in an efficient, accurate, and user-friendly manner. Software and test dataset are available at http://sunlab.lihs.cuhk.edu.hk/methy-pipe/.

## Introduction

DNA methylation is a biochemical process that predominantly involves the addition of a methyl group to cytosine nucleotides by DNA methyltransferases. This process plays an important role in the regulation of gene expression in both normal and dysfunctional cells [1]. Recently, with the advancement of massively parallel sequencing technologies, it has become feasible to explore DNA methylation in a genome-wide manner at single base resolution in a variety of biological systems with whole-genome bisulfite sequencing approach [2], [3], [4]; it requires the treatment of DNA with sodium bisulfite to convert Cytosines (Cs) into Uracils (Us), while methylcytosines remain unmodified. Since all Us are amplified by PCR as thymines (Ts), by comparing the modified DNA with the original sequence, the methylation state of the original DNA can be inferred by counting the number of cytosines and thymines at genomic cytosine sites.

Several library preparation protocols have been developed such as Reduced Representation Bisulfite Sequencing (RRBS) [5], in which only CpG dinucleotide within the CCGG sequence context can be studied using a methylation-insensitive restriction enzyme MspI. To overcome this limitation and gain genome-wide coverage for CpGs, other bisulfite sequencing protocols such as MethylC-Seq [3] and BS-Seq [2] have been developed. These two protocols mainly differ in their amplification procedures. Due to the simplicity of MethylC-seq protocol and the availability of commercial kits, it has recently been used for the whole genome DNA methylation studies in many tissues and samples [3], [4], [6], [7], [8], [9], [10]. Therefore, the demand for an integrative computational tool to analyze whole genome methylation data is increasing, especially for a tool that can satisfy multiple requirements (e.g., methylation-aware alignment, identification of Differentially Methylated Regions (DMRs), *etc.*) that are posed by different research focuses. Unfortunately, most of the existing tools cannot provide such a comprehensive spectrum of analysis. For example, some software packages are designed for bisulfite sequencing read alignment only [11], [12], [13], [14], others are for specific downstream analysis [15], [16]. To fill this gap, we implemented Methy-Pipe, an integrative bioinformatics software package that not only meets the core methylation data analysis demands but also provides a variety of analysis tools to facilitate the downstream analysis in an efficient and integrative manner.

## Implementation and Methods

### Overview of Methy-Pipe

**Figure 1:**
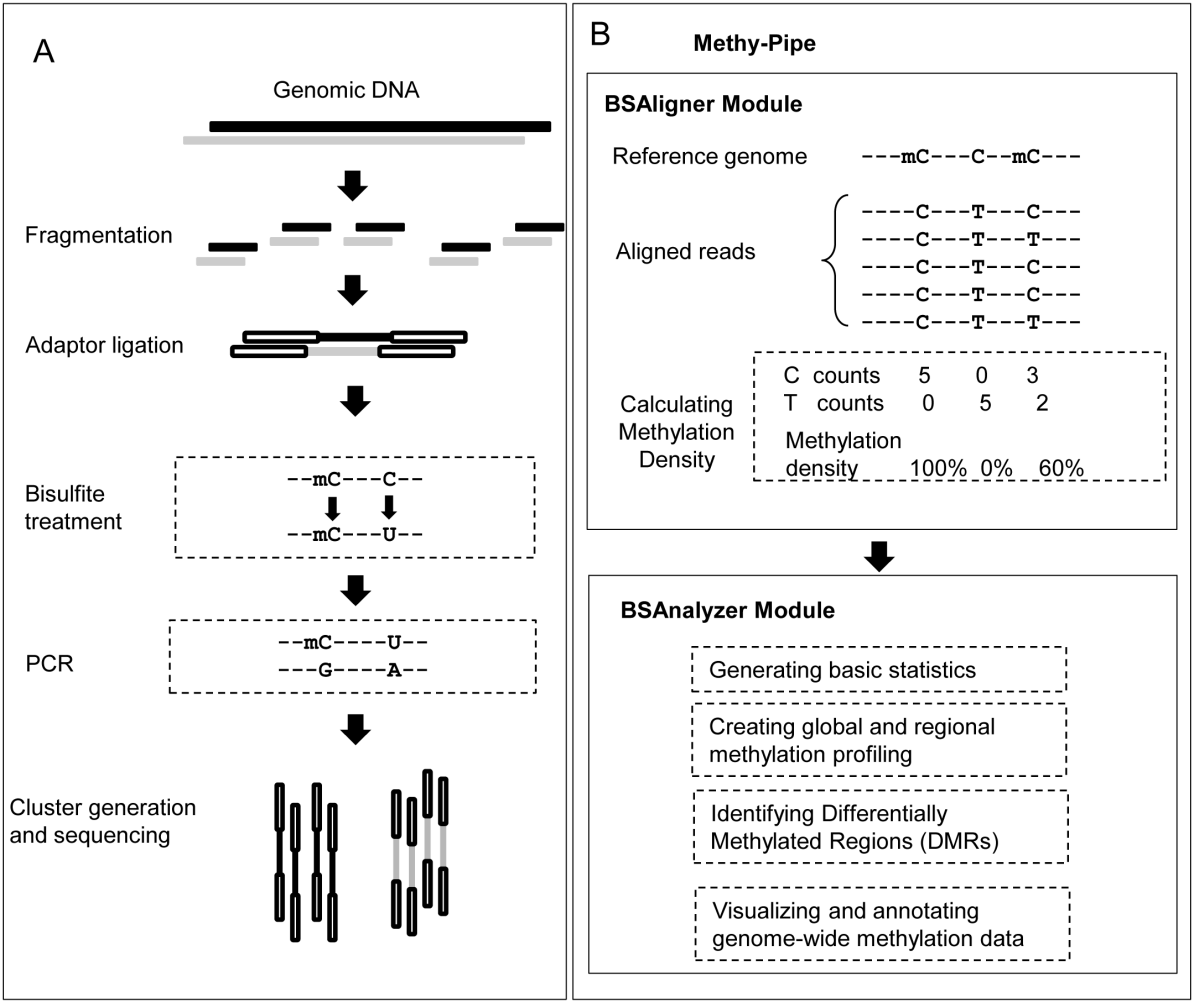
Schematic overview of MethyC-seq protocol and Methy-Pipe workflow. (A) The workflow of MethyC-seq library preparation and sequencing protocol. (B) The workflow and functional models of Methy-Pipe. DMRs: differentially methylated regions.

Methy-Pipe is designed to analyze the bisulfite sequencing data from the MethylC-Seq protocol (Figure 1A) [3]. The overall workflow of Methy-Pipe is illustrated in Figure 1B. Briefly, methylation data analysis is conducted through two consecutive software modules: ***(i)*** BSAligner module, a bisulfite sequencing read alignment module for data pre-processing and sequence alignment that is implemented based on 2BWT [17] source code (http://i.cs.hku.hk/2bwt-tools/downloads/2bwt-lib-v1.0.0-x84-64bit.tar.gz) and SOAP2 [18] source code (http://soap.genomics.org.cn/down/SOAPaligner-v2.20-src.tar.gz); ***(ii)*** BSAnalyzer module, a data analysis module implemented to provide a variety of functionalities to facilitate the downstream methylation data analysis. The major functions implemented in this module are: *(1)* to report the basic statistics and sequencing quality of the data; *(2)* to calculate the methylation level for any cytosine site and report genome-wide methylation profiles of the analyzed samples; *(3)* to identify DMRs for paired samples; and *(4)* to annotate and visualize the methylation data for data mining and easy interpretation. The details of the Methy-Pipe and the implementation of its functional modules will be further elaborated in the following sections.

### Input

The input data for Methy-Pipe consists of high-throughput bisulfite sequencing reads sequenced from either single or paired-end library prepared according to the MethylC-Seq protocol [3]; FASTQ format is required in which both the sequenced reads and their corresponding quality scores are stored in one text file.

### Bisulfite sequencing read alignment

**Figure 2:**
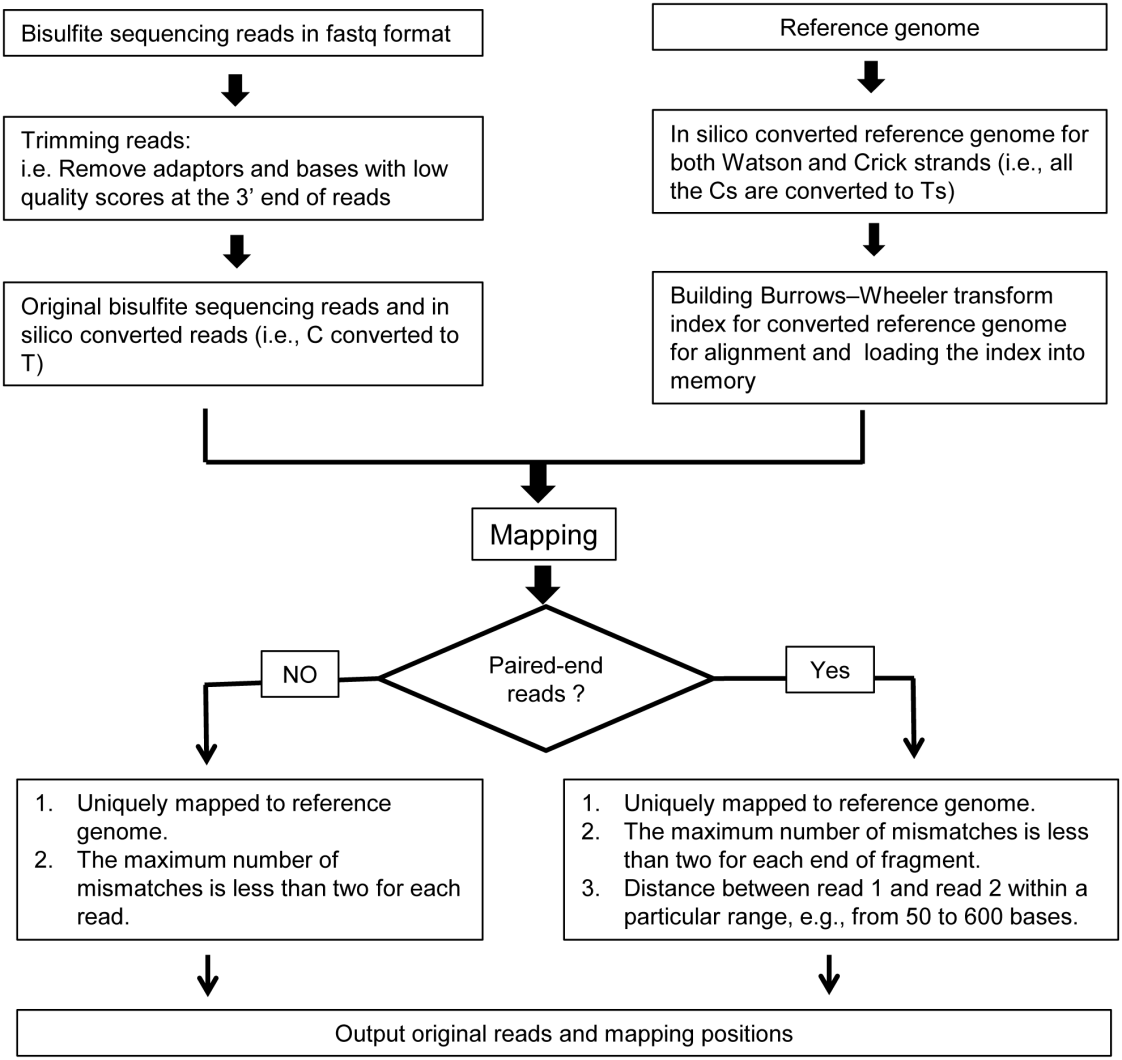
Principle of bisulfite sequencing read alignment by BSAligner. Firstly, the low-quality bases and sequenced adaptors at the 3′ ends of the reads are removed. The preprocessed reads are then mapped to C-to-T converted reference genomes whose Burrows-Wheeler Transform (BWT) indices are created and loaded to computing memory before executing alignment. Paired-end reads and single-end reads use different alignment strategies: (1) For single-end reads, they are mapped to reference genome by allowing at most 2 mismatches and only uniquely mapped reads are kept for further analysis; (2) For the paired-end reads, in addition to considering the number of mismatches and aligned hits, the insert size between the paired-end reads are also taken into account (e.g., from 50 to 600 bases); (3) The ambiguous reads that are mapped to both Watson and Crick strands are removed. Finally, the alignments are outputted in a text file which records the aligned chromosomes, positions, mismatches as well as sequencing qualities *etc.*

To align bisulfite sequencing reads back to the reference genome, we implemented BSAligner (Figure 2). Briefly, to trim the raw sequence reads, the following two filtering steps are applied to remove: ***(i)*** the sequencing adaptors; and ***(ii)*** low quality bases (i.e. bases with quality score <5) on read ends. The processed reads are then aligned to the *in* *silico* converted reference genomes. To prepare the *in* *silico* converted reference genomes for methylation awareness alignment, two C depleted reference genomes are built *in* *silico* by computationally converting all Cs to Ts in both Watson and Crick strands. Whole genome sequence indices of these two converted genomes are then created using Burrows-Wheeler transform (BWT) algorithm [17], [18]. During the alignment, BSAligner first loads those indices into the computer memory. Then, all Cs in the sequenced reads were replaced by Ts *in* *silico*. The pre-processed and converted reads were then aligned to the pre-converted reference genomes. After the alignment, first, we discard those reads that can be aligned back to both the Watson and Crick strands. Then the remaining *in* *silico* converted alignable reads are replaced by the original bisulfite sequencing reads and used for downstream methylation data analysis.

### Calculation of the methylation density (MD) level

To calculate the methylation density, we first count the total number of nucleotide C and T that overlap with each genomic cytosine site across the whole genome. If the sequenced fragment is so short that the sequenced read 1 and 2 overlap each other and the overlapped region covers the genomic cytosine sites, only one sequenced C or T with higher quality score will be counted. Then the MD can be calculated by the following equation:
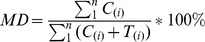



Where, in a given genomic region, n is the total number of cytosines, C_(i)_ is the total number of sequenced cytosines at the i^th^ cytosine position in the reference genome, suggesting the methylated event, and T_(i)_ is the total number of sequenced thymines at the *i* ^th^ position which is suggestive of unmethylated event. When n equals to 1, MD at a single-base resolution could be calculated.

### Identification of DMRs

**Figure 3:**
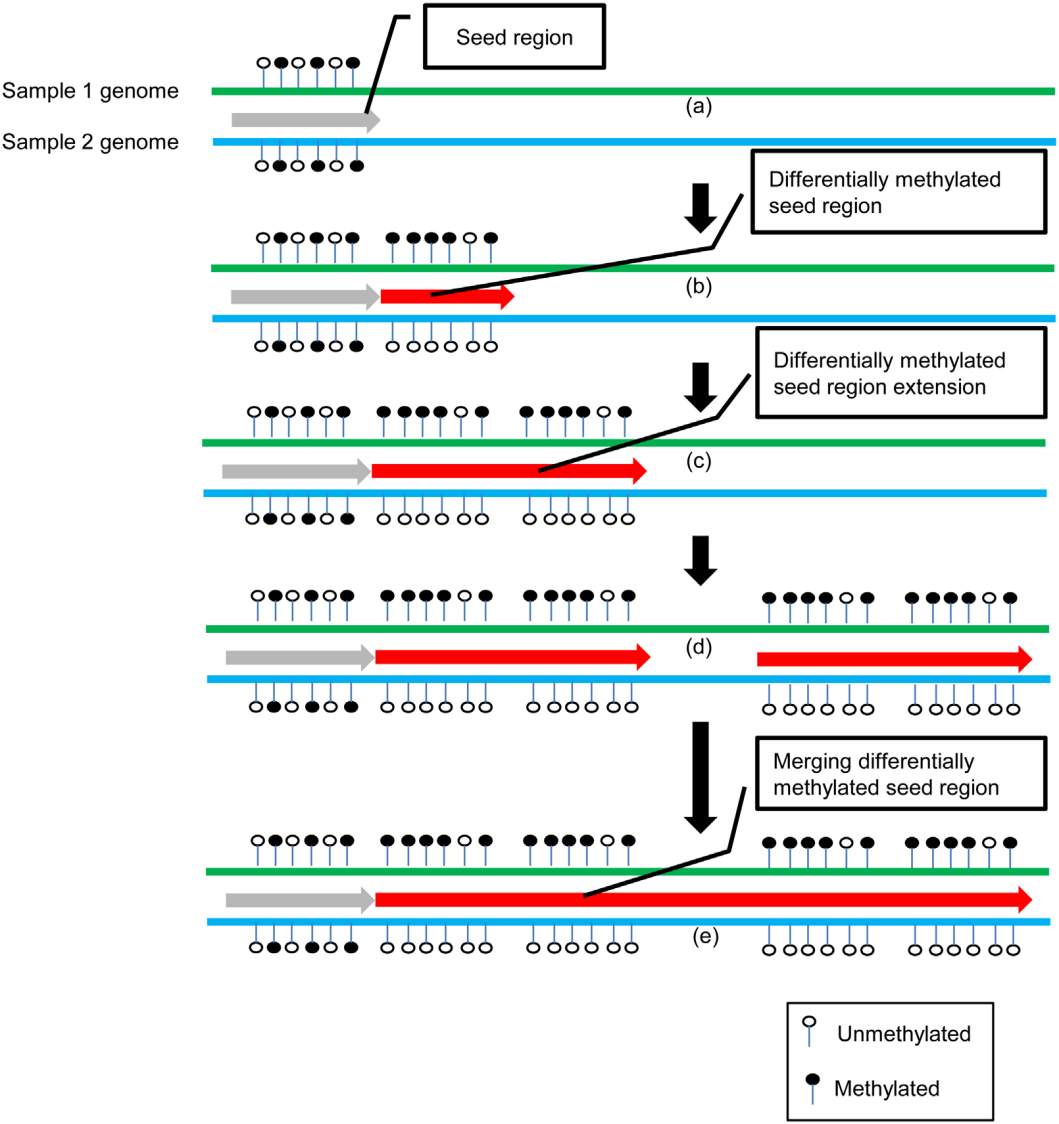
Principle of DMR detection by BSAnalyzer. (A) Firstly, starting from one end of the genome to search for a seed region (i.e., 500 bps) using a sliding window. (B) If the seed region is located, Mann-Whitney test will be used to test if the seed region is a differentially methylated seed region. (C) Two adjacent differentially methylated seed regions are merged into one extended seed region (seed region extension). (D) Two discontinued differentially methylated regions are further merged together if they are within a certain distance (e.g. less than 1000 bps) for further differential methylation test using χ2 test.

To identify DMRs genome-wide between two compared samples, a sliding window approach is implemented (Figure 3) with four key steps: ***(i)*** determining seed regions; ***(ii)*** identifying differentially methylated seed regions; ***(iii)*** differentially methylated seed region extension; and ***(iv)*** merging of adjacent differentially methylated seed regions. More specifically, to determine seed region, initially, a w-base (e.g., w = 500 bps) sliding window is applied from one end of the chromosomes of two compared samples. A w-base sliding window can be defined as a seed region if it meets the following criteria: (1) for both samples, the sliding window should contain at least *m* valid CpG sites (e.g., *m* = 5); (2) each valid CpG site should be covered by at least n bisulfite sequencing reads (e.g., n = 5). Otherwise this w-base window will be slid downstream with a s-base increment each time (e.g., s = 100) until the aforementioned criteria are satisfied.

Next, to identify differentially methylated seed region, the MD of each valid CpG site is calculated. Mann-Whitney U test is employed to test if the MDs of those valid CpG sites are statistically different (e.g., p-value<0.01) between two compared samples. If the test is statistically significant, this region is identified as a differentially methylated seed region.

To extend this region, we use the same approach to interrogate its adjacent downstream w*-*base window. If it is also a differentially methylated seed region, the two regions will be merged together. The same procedure will be repeated until the extended region is longer than *k* bases (e.g., k = 1000) or the adjacent region does not satisfy the criteria as a differentially methylated seed region.

Lastly, we merge the adjacent differentially methylated seed regions within 1000 bases of each other if they share similar methylation profile, i.e. ***(1)*** with similar methylation pattern in both samples. For example, both regions are more methylated in one sample than the other or vice versa; ***(2)*** with differences in MDs less than 10% in the same sample. Next, all qualified CpG sites within a merged differentially methylated region will be further subjected to χ^2^ test to assess if the proportion of the sequenced methylated cytosines over the total sequenced methylated and unmethylated cytosines is statistically different between two compared samples (default p-value≤0.01). The final merged differentially methylated seed regions with significant difference are considered as putative DMRs.

Notably, our algorithm is different from BSmooth [16] which requires biological replicates for DMR identification and is also different from MethylKit [19] which focuses on detecting differentially methylated cytosines (DMCs, rather than DMRs) when the biological replicates are absent.

### Implementation of Methy-Pipe

Methy-Pipe is implemented using Perl, R as well as C++. It is designed to run on x86_64 GNU/Linux platform. The data analysis performance can be enhanced by distributing multiple samples to different computing nodes using a Sun Grid Engine (SGE), for example, running on a Rocks cluster (http://www.rocksclusters.org).

## Results

To demonstrate the functionality and usage of Methy-Pipe, we applied it to a whole genome bisulfite sequencing dataset from our previous study [10]. In total, only 193 and 140 million paired-end bisulfite sequenced raw reads, which is equivalent to an average of ∼8x and ∼6x coverage, were used for the methylation data analysis for the maternal buffy coat and placenta sample, respectively.

### Methy-Pipe alignment module can accurately align the bisulfite sequencing reads

To show how Methy-Pipe can be used for bisulfite sequencing read alignment, we applied it to the aforementioned dataset with the functions implemented in *BSAligner* module (Figure 1B, Figure 2). Briefly, the bisulfite sequencing reads were pre-processed by filtering the low quality reads, *in* *silico* converted and aligned to the *in* *silico* converted reference genomes (see Methods). After the alignment, the following output files were created by Methy-Pipe: **(** ***i*** **)** the aligned reads stored in a text file (*.bsalign) with duplicated reads removed (Table S1); **(** ***ii*** **)** methylation call data in text files for both Watson and Crick strands. In these files, for each cytosine in the reference genome, the total number of the sequenced methylated cytosines (Cs) and unmethylated cytosines (Ts) as well as the sequence context are reported (Table S2). Notably, for both placenta and buffy coat samples, above 80% of bisulfite sequencing raw reads could be mapped back to the human reference genome (Table S3).

To further evaluate the performance of *BSAligner*, we tested if it can align the bisulfite sequencing reads to the reference genome in an efficient and accurate manner. To this end, we first computationally generated ∼1 million simulated 75 base bisulfite sequencing paired-end reads from the lambda genome. The insert sizes of those simulated paired-end reads ranged from 75 to 600 bases. The methylation level of all the cytosines in CpG dinucleotides are set to be 100%. Using 20 cores on an Intel Xeon 2.80 GHz CPU, it took approximately 25 s to complete the entire alignment, suggesting BSAligner is a very efficient alignment tool. The resultant alignment accuracy is 99.9%, with a mappability of 99.9%, suggesting an excellent performance.

**Figure 4:**
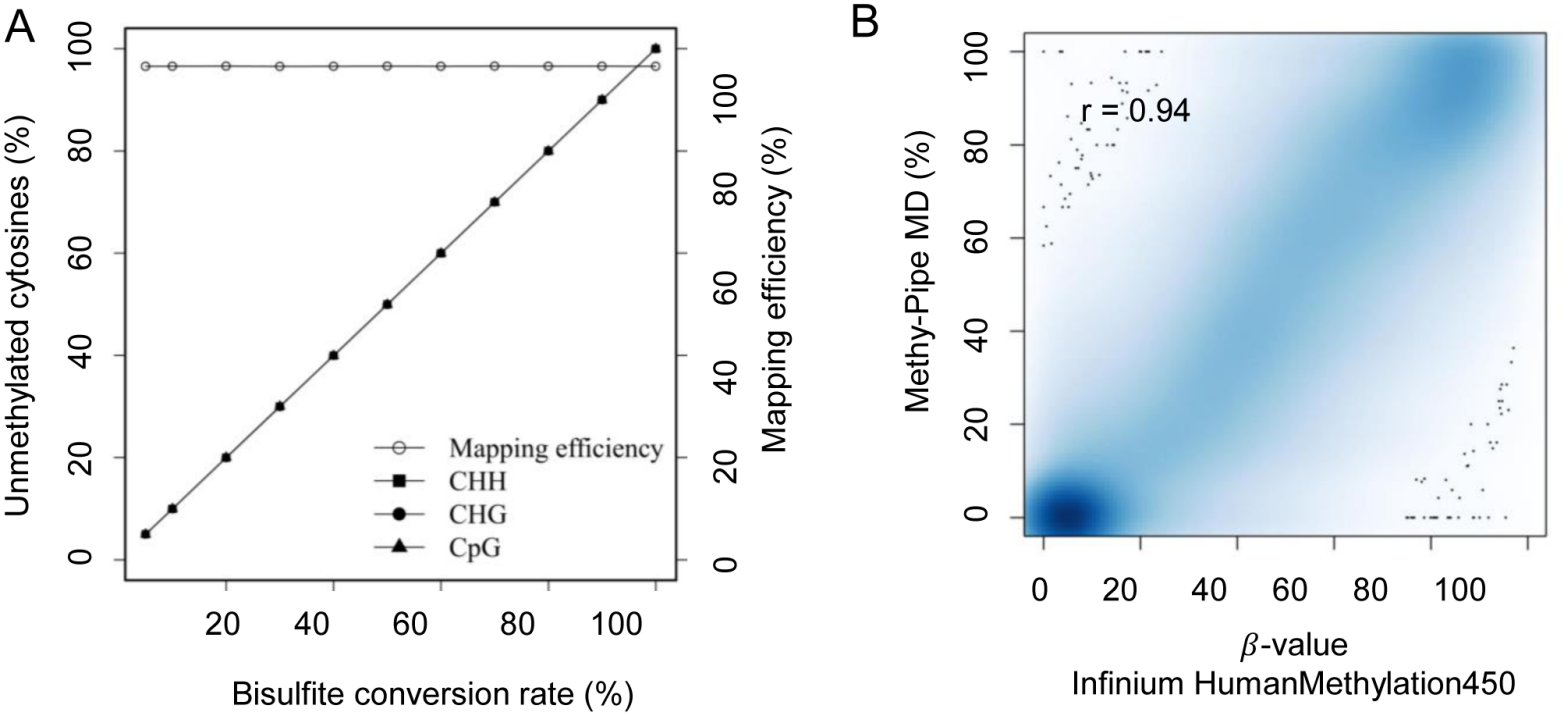
Performance evaluation of Methy-Pipe. (A) A total of 1 million reads (75 bases) were randomly simulated with different rates of bisulfite conversion and aligned to human (GRCh37/hg19) genome. Methy-Pipe accurately detected various simulated methylation levels at a constant mapping efficiency which is not affected by the sequence context. The H (in CHG and CHH) denotes C, T, or A. (B) Density scatter plots are plotted to measure the correlation between the MDs from Methy-Pipe and the *β* value from Infinium Human Methylationa450 array for CpG loci that can be integrated by both sequencing and array platforms from Placenta.

Next, to test the accuracy of Methy-Pipe alignment for the bisulfite sequencing reads with different levels of methylations, we obtained a simulated dataset from human genome that contains ∼1 million bisulfite sequencing paired-end reads with the simulated methylation level ranging from as low as 5% to 100%. By aligning these reads using BSAligner, Methy-Pipe can accurately detect the simulated methylation states of cytosines in any sequence context while the mapping efficiency is completely unaffected (Figure 4A).

Lastly, we compared Methy-Pipe’s sequencing alignment module, BSAligner, with Bismark [12], a bisulfite read alignment program outperforming most of the other aligners. Based on 1 million *in* *silico* simulated methylation data, BSAligner outperformed Bismark in terms of the computation time (Table S4). For Bismark [16], it took 21 minutes and 67 minutes of CPU time using Bowtie1 [20] and Bowtie2 [21] respectively, while it only took 16 minutes for BSAligner with a comparable alignment accuracy (Table S4). In addition, our whole pipeline enables the analyses to be distributed to different computing nodes in a parallel manner based on SGE platform, which dramatically enhances the speed of analyses for large-scale methylation studies in orders of magnitude.

### Methy-Pipe can Accurately Quantify MDs of the Regions of Interests

To accurately calculate MDs (see Methods) is the first step towards the quantification of methylation data. To access if MD, calculated by Methy-Pipe based on the whole genome bisulfite sequencing data, can represent the methylation state of each CpG site accurately, we compared it with the methylation state measured by another independent platform, Illumina Infinium HumanMethylation450 BeadChip [10], which includes more than 480 K CpG sites for interrogation. Two tissue samples were run on both platforms, one with placenta tissue and the other with a paired maternal buffy coat sample. For the calculation of MD with Methy-Pipe, each CpG loci on HumanMethylation450 array needs to be covered by at least 10 aligned reads, which resulted in 310,319 and 267,946 CpG loci for comparisons in the placenta and buffy coat sample, respectively. To measure the methylation state of CpG loci, the Methylation Module (v1.9.0) of the GenomeStudio (v2011.1) software was used. The methylation state for individual CpG site was measured by the beta value (β), which is calculated using the ratio of fluorescence intensities between methylated (M) and unmethylated (U) alleles in the equation 2 below:
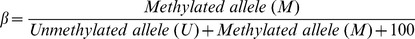



As a result, there is a good concordance between the MDs obtained from Methy-Pipe and the values obtained from Illumina Infinium HumanMethylation450 array for CpG sites analyzed. The Pearson correlation coefficient of 0.94 for placenta sample (Figure 4B) indicates that the MDs calculated by Methy-Pipe and values from Infinium HumanMethylation450 array are consistent in measuring the DNA methylation states across the queried CpG loci.

### Basic statistics of methylation data reveals the overall data quality

**Figure 5:**
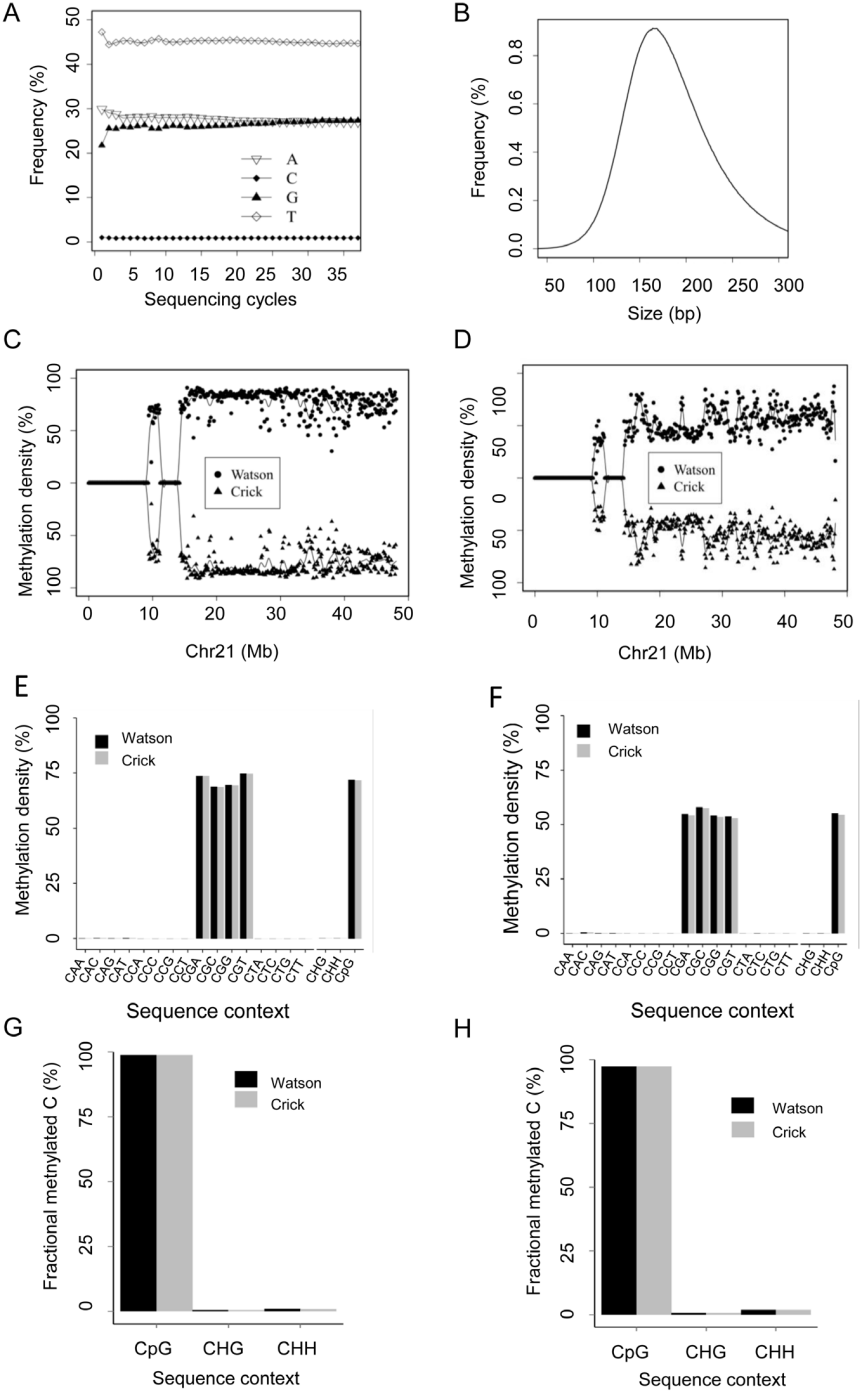
Summary of Methy-Pipe results from BSAnalyzer module. (A) The plot of the base (A, C, G, T) frequency at each sequencing cycle. X-axis indicates the sequencing cycle. Y-axis indicates the base frequency. (B) The length distribution of the insert size of a paired-end bisulfite sequencing library. X-axis represents the insert size. Y-axis represents the percentage of insert with the indicated size. (C, D) Whole genome methylation profiling with fixed window approach for buffy coat sample (C) and placenta sample (D). Dots on the top are for the Watson strand and triangles on the bottom are for the Crick strand. (E, F) Whole genome methylation profiling within different sequence contexts. MDs at different sequence contexts, namely CAA, CAC, CAG, CAT, CCA, CCC, CCG, CCT, CGA, CGC, CGG, CGT, CTA, CTC, CTG, CTT, are calculated for buffy coat (E) and placenta (F), respectively. (G, H). The fractions of the methylated cytosines are calculated for 3 different sequence contexts for buffy coat (G) and placenta (H), respectively. Fractional methylated C is calculated as the proportion of the methylated cytosines at a particular sequence context over total methylated C sequenced. The results indicate that most of the methylated cytosines are from CpG dinucleotides, i.e. CGA, CGC, CGG and CGT. The H in CHG, CHH represents A, C, or T.

After the alignment and MD calculation, it is necessary to provide the basic statistics of the sequenced methylation data in order to have a global overview about the overall data quality. To achieve this, Methy-Pipe generated a HTML file, which summarizes basic statistics and quality control information from the aligned bisulfite sequencing data, with the HTML links coded in the file for users to navigate. In this HTML file, the following useful information are included: total number of sequenced fragments from each sample, mappability, duplication rate, the percentage of cytosines or cytosines in the context of CpG dinucleotides covered by at least one sequenced reads, average sequencing depth, overall methylation density of different sequencing context (i.e., CpG, CHG and CHH; H represents A, C, or T), and bisulfite conversion rate estimated by the spiked lambda genome if available (Table S3). In addition, two plots were generated to assess the experimental quality: ***(i)*** a plot of base compositions of four nucleotides at each sequencing cycle, in which a high percentage of T and low percentage of C are expected due to the base conversion involved in the bisulfite sequencing procedure (Figure 5A). A deviation from this trend usually indicates a sub-optimal sequencing condition (e.g. incomplete bisulfite conversion, *etc.*); ***(ii)*** a plot of the distribution of the insert size for the paired-end sequencing. This can help to monitor if the insert size of the sequencing library is as expected or not (Figure 5B).

### Whole genome methylation profiling can reveal the important biological features of the studied samples

To gain insights into the methylation states of the samples, Methy-Pipe provides two ways to obtain a genome-wide methylation profile for a given sample: *(i)* to generate genome-wide methylation profiles using MDs of fixed windows across the whole genome to visualize the MDs in a scatter plot. In the scatter plot, each dot represents a genomic region with a fixed length (i.e. 100 kb). The MDs of these fixed windows are plotted against their genomic locations in the reference genome (Figure 5C, D). If comparing the methylation level of multiple samples, a Circos plot [22] can also be generated; ***(ii)*** to provide MDs for cytosines under different sequence contexts (i.e. CGH, CHG, and CHH, where H = A, C or T) (Figure 5E, F). When applying Methy-Pipe to our dataset, as demonstrated for chromosome 21, the placenta is hypomethylated and characterized with more fluctuating methylation patterns (Figure 5C, D). Further studies showed that the MDs in the placenta genome are lower than that in the maternal buffy coat, which is consistent with reports on the hypomethylated nature of placental tissues. Furthermore, for both samples, nearly all of the methylation occurred on CpG sites with much higher MDs compared to other sequence contexts (Figure 5G, H), suggesting that the majority of methylation events in these two tissues occur in the sequence context of CpG.

### Methylation profiling for genomic regions of interest provides tools to study DNA methylation at different resolutions

**Figure 6:**
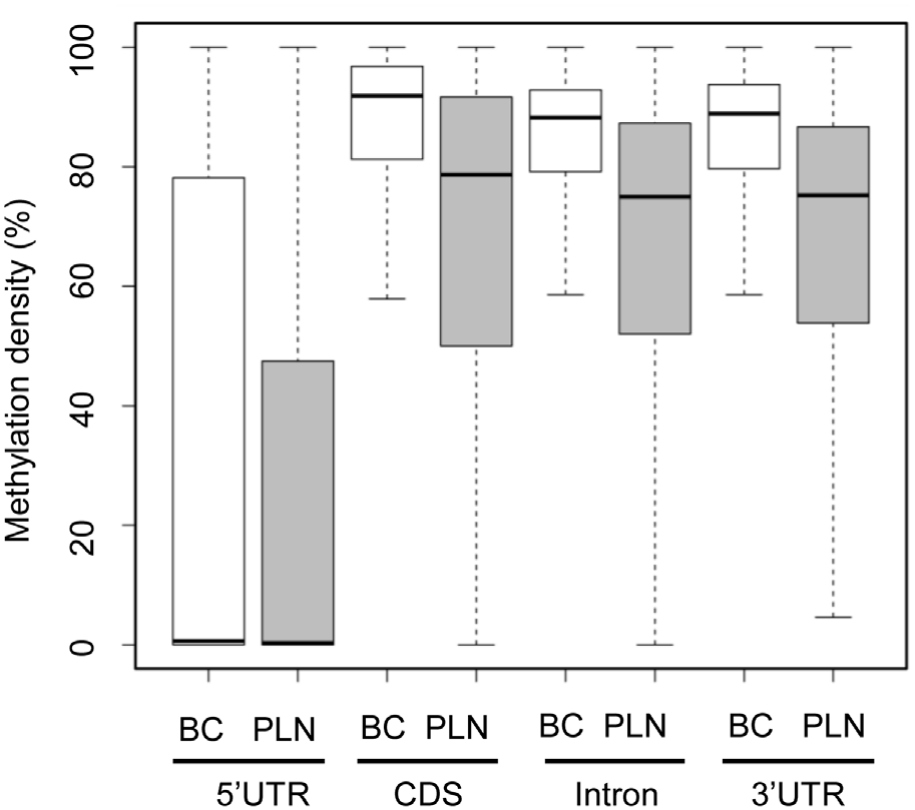
Methylation profiling for different genomic regions. BC: Buffy coat; PLN: Placenta.

In addition to investigating the methylation states at whole genome level, studying the methylation profiles around specific genomic regions can also shed light on how DNA methylation affects gene expression. For example, MDs around transcription start sites (TSSs) are commonly correlated with the expression levels of epigenetically regulated genes [1]. On the other hand, MDs within the repeat regions (e.g. LINEs, SINEs, *etc.*) were found to be hypo-methylated in a genome-wide manner in various cancers [6], [23]. Methy-Pipe is comprised of a utility program to calculate the MD of any genomic region of interest provided by the user. The output is a table that reports the methylation states of each cytosine within that region (Table S5). In addition, Methy-Pipe can also present the results as box plots (Figure 6). We thus applied Methy-Pipe to our experimental dataset and calculated the methylation profiles in the 5′UTR, coding sequences (CDS) and intron regions of all annotated human protein coding genes. Results revealed the MDs around 5′ UTR regions are sharply reduced for both placenta and buffy coat samples. This observation is consistent with what has been documented in other studies [7]. Further comparison of MDs of the two samples in genomic regions described above revealed distinct methylation patterns. A significant higher level of MDs was detected in gene bodies when compared to the 5′UTR regions. Interestingly, CDS has the highest MDs followed by intron and 3′ UTR regions. These results demonstrate the variety of functions that Methy-Pipe allows the users to mine out the biological significance hiding behind the complicated methylation data.

### Methy-Pipe can accurately identify DMRs with comparatively low sequencing depth

The identification of DMRs is one of the major goals of methylation data analysis. Methy-Pipe implements a DMR identification algorithm in BSAnalyzer module (see Methods). To demonstrate how to use this algorithm in Methy-Pipe for the identification of DMRs, we applied it to the placenta and buffy coat datasets. As a result, two files were created as the outputs of the identification of DMRs: ***(i)*** A DMR list file reporting the detailed information of the identified DMRs, such as genomic locations, methylation states (hypomethylated or hypermethylated), cytosine and thymine counts, and the *p*-values for the statistical test, *etc.* (Table S6); ***(ii)*** A DMR annotation file providing the information on the neighboring gene(s) for each DMR (Table S7).

**Figure 7:**
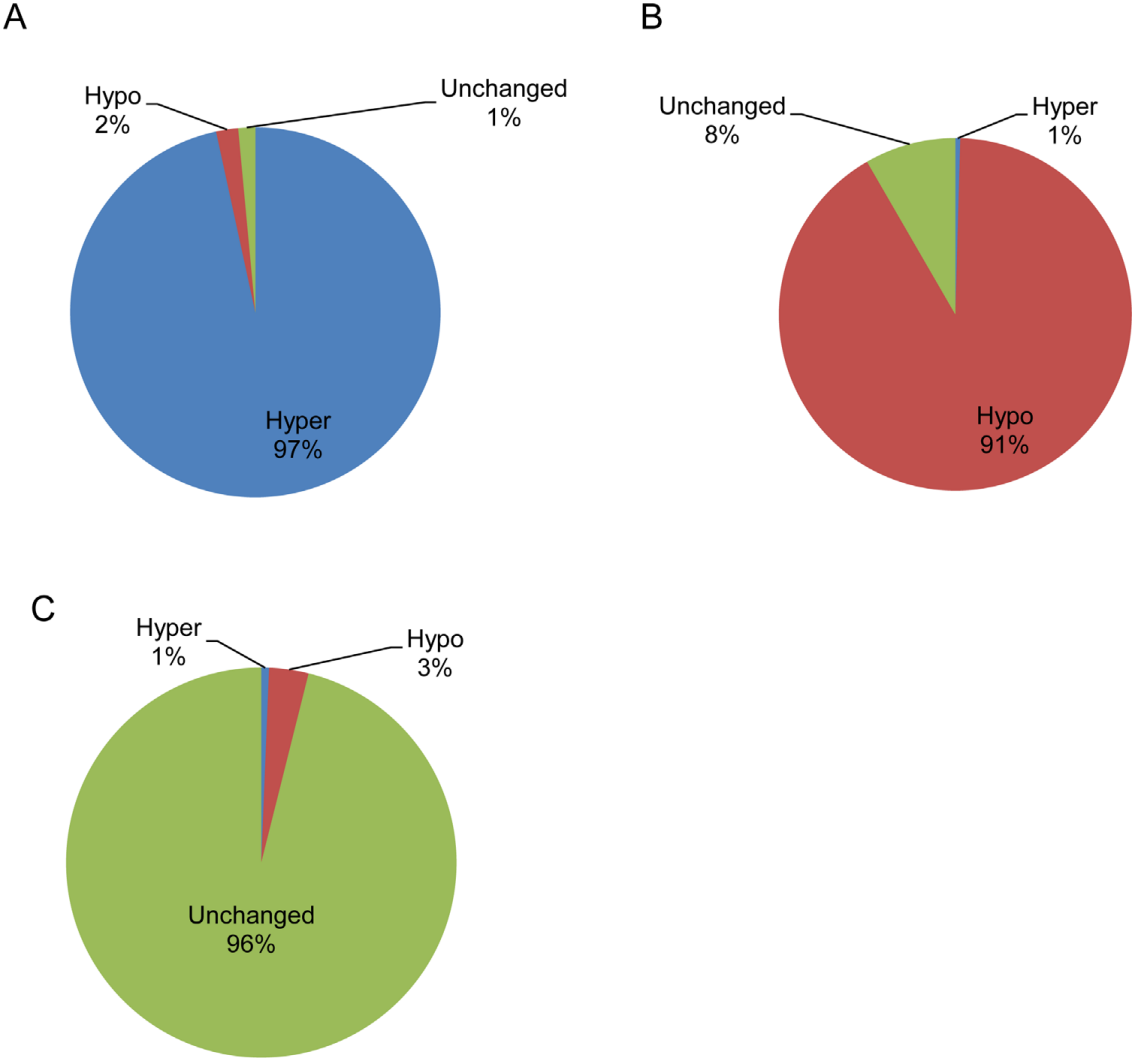
Performance evaluation of Methy-Pipe DMR identification algorithm using methylation data from Infinium HumanMethylation450 array. (A) The proportion of hypermethylated regions identified by the array platform also detected by the Methy-Pipe. (B) The proportion of hypomethylated regions detected in array also identified by the Methy-Pipe. (C) Comparison of the methylation level unchanged regions between the array platform and Methy-Pipe. Hypo: hypomethylated regions. Hyper: hypermethylated regions. Unchanged: unchanged regions.

To evaluate the performance of DMR algorithm implemented in BSAnalyzer module, using the evaluation strategies adapted from BSmooth [16], we also established sets of genomic regions of hypo- and hyper-methylated DMRs as well as the regions without methylation state changes by comparing placenta to its paired buffy coat tissue samples as “gold-standard” references through Illumina Infinium HumanMethylation450 methylation data with the following criteria [16]: ***(i)*** the values was first calculated as the mean value of a group of the probes on HumanMethylation450 array within 500 bp window; ***(ii)*** the hypermethylated regions were defined by the difference of mean values between placenta and buffy coat larger than 25%; ***(iii)*** the hypomethylated regions were defined by the difference of mean values between the two samples larger than 25% in a reverse direction; ***(iv)*** The unchanged regions were defined by the difference of mean values between the two samples within 3%. Based on the above definition, 676, 2,650 and 9,249 regions were identified as hypermethylated, hypomethylated and unchanged regions. When comparing the above regions with the corresponding regions identified from Methy-Pipe using default parameter settings (*p*<0.01 for hypermethylated or hypomethylated regions, *p*>0.25 for unchanged regions) from data with comparatively low sequencing depth (∼8x and 6x coverage for buffy coat and placenta, respectively), we could achieve 97%, 91%, and 96% accuracy for the detection of hypermethylated, hypomethylated, and unchanged regions (Figure 7). These results suggest that the Methy-pipe can accurately identify DMRs with comparatively low sequencing depth.

**Figure 8:**
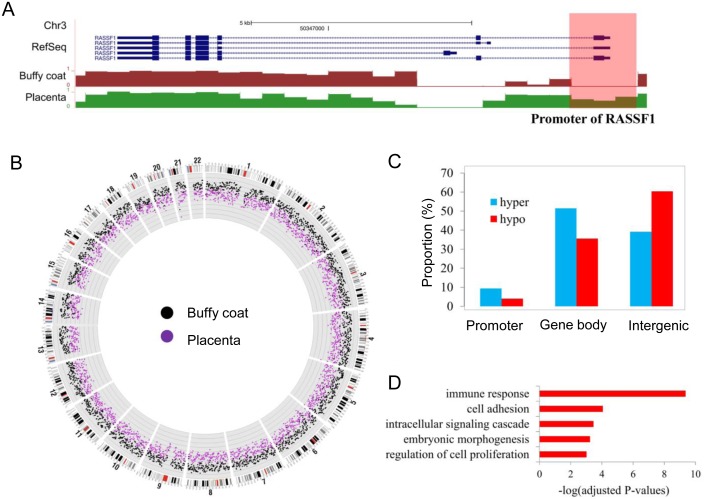
Biological insights revealed by Methy-Pipe. (A) A snapshot of methylation density in promoter and gene body regions of RASSF1A gene. A DMR in promoter region by comparing buffycoat and placenta tissue using Methy-Pipe is highlighted. (B) Genome-wide methylation profiling of the global methylation pattern for maternal buffy coat and the placenta samples. The range of MD shown is from 0% (innermost) to 100% (outermost) and the distance between two lines is 10%. (C) The distribution of DMRs identified by Methy-Pipe across different genomic features. (D) Gene ontology analysis of hypermethylated regions in placenta. Hypo: hypo-methylated. Hyper: hyper-methylated.

To further evaluate the quality of the identified DMRs, we selected a subset of the above identified DMRs where the MDs of the maternal buffy coat DNA were either ≤20% or ≥80% and the MDs in the placenta were significantly differed by at least 20% from those of the maternal buffy coat. Such criteria were selected so that molecular assays could be designed to differentially detect the placenta-derived and maternal buffy coat-derived DNA sequences in maternal plasma. Using this method, we identified 17,924 hyper-methylated and 164,846 hypo-methylated DMRs. Furthermore, a utility program in Methy-Pipe was used to annotate these DMRs to the closest genes (i.e., within 2 kb of the upstream of transcription start site of the closest protein coding gene). As a result, 1,688 hypermethylated and 6,793 hypomethylated DMRs were associated with the known annotated genes. For example, the promoter of RASSF1A gene is more hypermethylated in placenta compared with maternal buffy coat (Figure 8A), which is in agreement with the previous report [24]. Further analysis for those associated DMRs indicates that even though the placenta was shown to be pervasively hypomethylated compared with the maternal buffy coat in a genome-wise manner (Figure 8B), the hypermethylated DMRs are more enriched within the promoter regions of the associated genes when compared to hypomethylated ones (Figure 8C). This finding suggests that the hypermethylation might potentially play some roles in gene regulation of the placenta. In addition, Gene Ontology (GO) analysis [25], [26] of those genes associated with hypermethylated DMRs revealed that a significant number of them are relevant to cell adhesion and embryonic organ morphogenesis (Figure 8D), which is in line with the previous study on the placenta epigenetics [27]. These results indicate that the DMRs identified by Methy-Pipe may have biological functions and could be potential targets for exploring the possibilities of clinic applications in the prenatal diagnosis.

### Computational cost of Methy-Pipe

To further demonstrate the computational cost of Methy-Pipe, we also tested it on 10 million 75 bp paired-end bisulfite sequencing reads. It took 33 minutes to complete the whole analysis with peak memory usage of 25 GB based on an Intel Xeon X5675 CPU using 20 cores.

## Discussion

In this study, we designed and implemented Methy-Pipe, an integrated whole genome bisulfite sequencing data analysis pipeline. It not only fulfills the core data analysis requirements such as bisulfite-treated sequencing read alignment, methylation level inference, and DMR identification but also provides a variety of utility programs to further annotate and visualize the resulting methylation data. Using real datasets from human placenta and maternal buffy coat samples, we demonstrated that Methy-Pipe can efficiently and accurately analyze the whole genome bisulfite sequencing data. Thus, this new pipeline would facilitate us to develop next generation sequencing based diagnostic approaches based on the DNA methylation marker in many research areas of medical genomics such as prenatal diagnosis [10] and cancer detection [6].

When compared with many previously reported whole genome bisulfite sequencing data analysis software packages, Methy-Pipe appears to demonstrate more functionality and is easier to use. First, it integrates the core and the downstream data analysis modules into one package so that the end user can explore the biological significance of methylation. In addition, Methy-Pipe can take advantage of the high-performance computing clusters by utilizing SGE to parallelize data analyzes, which could dramatically speed up the analysis of bisulfite sequencing data that is normally huge and demands intensive computing power.

Our BSAligner allows efficient alignment of sequencing reads. Compared to the majority of aligners designed for bisulfite sequencing data alignment, its performance has been greatly improved by integrating quality control filters before the read alignment is carried out. First, low quality bases of the two ends of the sequenced reads can be filtered, which decreases methylation call errors. Second, sequence adaptors can also be filtered to reduce the adaptor contamination for the short reads during the methylation inference. In addition, BSAligner adopts a methylation unbiased approach, in which any available cytosine in the sequenced read after bisulfite treatment and all cytosines in the reference genome are converted into thymines before the alignment. In the BSAligner, it can directly map the converted bisulfite reads to converted reference genome using the BWT algorithm, thus eliminating the time consuming step of converting all Cs to Ts during the alignment. As a result, the performance of BSAligner is better than that of Bismark [12] which is noteworthy as Bismark has been shown to outperform many previously reported mapping programs, including BSMAP [14], BS Seeker [11], and MAQ [28] in terms of the ability for paired-end read alignment and running time.

## Supporting Information

Table S1 The example output of BSAligner.
(DOCX)

Table S2 The example output of methylation call by Methy-Pipe.
(DOCX)

Table S3 The example output of basic statistics of methylation data reported by Methy-Pipe.
(DOCX)

Table S4 The performance comparison between BSAligner and Bismark.
(DOCX)

Table S5 The example output of methylation densities for the regions of interest.
(DOCX)

Table S6 The example list of identified DMRs.
(DOCX)

Table S7 The example list of DMRs annotated with the closest genes.
(DOCX)
