# Supplementary material for: Methy-Pipe: An Integrated Bioinformatics Pipeline for Whole Genome Bisulfite Sequencing Data Analysis
Source: PLoS One. 2014 Jun 19;9(6):e100360. doi: 10.1371/journal.pone.0100360 (PMC4063866; doi:10.1371/journal.pone.0100360)
Supplement: Table S1 — The example output of BSAligner. (DOCX) [file pone.0100360.s001.docx]

**Table S1.** The example output of BSAligner

| **Read ID** | **Read** | **Quality** | **Hits** | **Read** | **Read length** | **Strand** | **Chr** | **Position** | **No. of mismatches** | **Mismatch tracking** | **CIGAR string** | **Alignment tracking** | **Watson/Crick** |
| --- | --- | --- | --- | --- | --- | --- | --- | --- | --- | --- | --- | --- | --- |
| HiSeq:7:1101:1232:1894#0/1 | AGAAT… | BJPJ`… | 1 | a | 62 | + | chr4 | 115754150 | 0 |  | 62M | 62 | C |
| HiSeq:7:1101:1232:1894#0/2 | AATAG... | fehd… | 1 | b | 62 | - | chr4 | 115754213 | 1 | G->9T24 | 62M | 9G52 | C |
| HiSeq:7:1101:1127:1949#0/1 | GGATA... | JJ\c`… | 1 | a | 62 | + | chr1 | 29390995 | 0 |  | 62M | 62 | W |
| HiSeq:7:1101:1127:1949#0/2 | GGATA... | ^[a[f… | 1 | b | 62 | - | chr1 | 29390995 | 0 |  | 62M | 62 | W |
