# Supplementary material for: Methy-Pipe: An Integrated Bioinformatics Pipeline for Whole Genome Bisulfite Sequencing Data Analysis
Source: PLoS One. 2014 Jun 19;9(6):e100360. doi: 10.1371/journal.pone.0100360 (PMC4063866; doi:10.1371/journal.pone.0100360)
Supplement: Table S2 — The example output of methylation call by Methy-Pipe. (DOCX) [file pone.0100360.s002.docx]

**Table S2.** The example output of methylation call by Methy-Pipe

| **Chr** | **Position** | **Base in reference** | **Total depth** | **Cytosine counts** | **Thymine counts** | **Sequence context** |
| --- | --- | --- | --- | --- | --- | --- |
| chr1 | 11310 | c | 1 | 0 | 1 | c:a:g |
| chr1 | 11315 | c | 1 | 0 | 1 | c:c:c |
| chr1 | 11316 | c | 1 | 0 | 1 | c:c:t |
| chr1 | 11317 | c | 1 | 0 | 1 | c:t:c |
| chr1 | 11319 | c | 1 | 0 | 1 | c:t:t |
