# Supplementary material for: Methy-Pipe: An Integrated Bioinformatics Pipeline for Whole Genome Bisulfite Sequencing Data Analysis
Source: PLoS One. 2014 Jun 19;9(6):e100360. doi: 10.1371/journal.pone.0100360 (PMC4063866; doi:10.1371/journal.pone.0100360)
Supplement: Table S3 — The example output of basic statistics of methylation data reported by Methy-Pipe. (DOCX) [file pone.0100360.s003.docx]

**Table S3.** The example output of basic statistics of methylation data reported by Methy-Pipe

|  | **Buffy coat** | | **Placenta** | |
| --- | --- | --- | --- | --- |
|  | **Watson** | **Crick** | **Watson** | **Crick** |
| **No. total fragments** | 192741960 | | 140721077 | |
| **No. mapped fragments^a^** | 79557779 | 78328115 | 58805365 | 58021641 |
| **Mappability (%)** | 41 | 41 | 42 | 41 |
| **Total mappability (%)** | 82 | | 83 | |
| **Whole genome depth** | 3.98 | 3.92 | 2.94 | 2.9 |
| **Nonduplicated fragments** | 77447909 | 76579954 | 56230014 | 55649794 |
| **Duplicated fragments^a^** | 2109870 | 1748161 | 2575351 | 2371847 |
| **Duplication rate (%)** | 2.652 | 2.23184 | 4.37945 | 4.08787 |
| **Lambda conversion Rate(%)^b^** | 99.98 | 99.98 | 99.97 | 99.97 |
| **Genomic C seq C count^c^** | 97378919 | 96061615 | 64083949 | 63277106 |
| **Genomic C seq T count^d^** | 1.623E+09 | 1.607E+09 | 1.545E+09 | 1.531E+09 |
| **Genomic C cov (%)** | 55.1429 | 55.0585 | 56.4516 | 56.3319 |
| **Genomic C seq depth** | 2.941 | 2.9111 | 2.75 | 2.7252 |
| **Genomic C met Density(%)** | 5.6593 | 5.6399 | 3.983 | 3.9685 |
| **CpG seq C count** | 96186480 | 94931887 | 62363394 | 61607602 |
| **CpG seq T count** | 48922248 | 44941811 | 68289813 | 64024731 |
| **CpG cov(%)** | 77.1982 | 77.1209 | 75.5186 | 75.3899 |
| **CpG seq depth** | 5.1859 | 4.9988 | 4.6693 | 4.4898 |
| **CpG met density(%)** | 66.2858 | 67.8697 | 47.732 | 49.038 |
| **CHG seq C count** | 370303 | 359356 | 474788 | 459921 |
| **CHG seq T count** | 495978226 | 490426567 | 440370021 | 434592885 |
| **CHG cov(%)** | 67.4977 | 67.3902 | 66.9569 | 66.8171 |
| **CHG seq depth** | 4.0071 | 3.9622 | 3.559 | 3.5123 |
| **CHG met density(%)** | 0.0746 | 0.0732 | 0.1077 | 0.1057 |
| **CHH seq C count** | 822136 | 770372 | 1245767 | 1209583 |
| **CHH seq T count** | 1.078E+09 | 1.072E+09 | 1.036E+09 | 1.033E+09 |
| **CHH cov(%)** | 52.056 | 51.9749 | 54.1621 | 54.0445 |
| **CHH seq depth** | 2.584 | 2.568 | 2.4839 | 2.4752 |
| **CHH met density(%)** | 0.0762 | 0.0718 | 0.1201 | 0.117 |

seq, sequenced; H refers to A, C or T nucleotide sequence; cov, coverage; met, methylation.

^a^refers to paired reads with identical start and end coordinates.

^b^lambda DNA was spiked into each sample before bisulfite conversion. The lambda conversion rate refers to the proportion of cytosine nucleotides that remain as cytosine after bisulfite conversion and is used as an indication of the rate of successful bisulfite conversion.

^c^refers to the number of cytosine nucleotides present in the reference human genome and remaining as a cytosine sequence after bisulfite conversion.

^d^refers to the number of cytosine nucleotides present in the reference human genome and coverted to a thymine sequence after bisulfite conversion.
