# Supplementary material for: Methy-Pipe: An Integrated Bioinformatics Pipeline for Whole Genome Bisulfite Sequencing Data Analysis
Source: PLoS One. 2014 Jun 19;9(6):e100360. doi: 10.1371/journal.pone.0100360 (PMC4063866; doi:10.1371/journal.pone.0100360)
Supplement: Table S4 — The performance comparison between BSAligner and Bismark. (DOCX) [file pone.0100360.s004.docx]

**Table S4.** The performance comparison between BSAligner and Bismark

|  | **Multiple threads** | | **CPU time (min)** | | **Mappablity** | | **Accuracy** | |
| --- | --- | --- | --- | --- | --- | --- | --- | --- |
| BSAligner | Yes | 16 | | 97% | | 99% | |  |
| Bismark (bowtie1) | No | 21 | | 97% | | 99% | |  |
| Bismark (bowtie2) | Yes | 67 | | 96% | | 99% | |  |

1 million bisulfite sequencing paired-end reads simulated by Sherman with insert size from 75 nt to 600 nt (<http://www.bioinformatics.babraham.ac.uk/projects/sherman/>). Since the conversion rate wouldn’t affect both Bismark [[1](#_ENREF_1)] and BSAligner, we only chose the parameter of conversion rate for the bisulfite sequencing read simulation at 10% in this test. The other parameters used in Sherman program corresponded to the default values.

1. Krueger F, Kreck B, Franke A, Andrews SR (2012) DNA methylome analysis using short bisulfite sequencing data. Nat Meth 9: 145-151.
