# Supplementary material for: Methy-Pipe: An Integrated Bioinformatics Pipeline for Whole Genome Bisulfite Sequencing Data Analysis
Source: PLoS One. 2014 Jun 19;9(6):e100360. doi: 10.1371/journal.pone.0100360 (PMC4063866; doi:10.1371/journal.pone.0100360)
Supplement: Table S5 — The example output of methylation densities for the regions of interest. (DOCX) [file pone.0100360.s005.docx]

**Table S5.** The example output of methylation densities for the regions of interest

| **Chr** | **Start** | **End** | **Description** | **Cytosine counts** | **Thymine counts** | **Methylation density** |
| --- | --- | --- | --- | --- | --- | --- |
| chr3 | 184375797 | 184376100 | AluSx,SINE/Alu | 7 | 1 | 87.5 |
| chr7 | 29320335 | 29320416 | MIRb,SINE/MIR | 0 | 1 | 0.0 |
| chr14 | 45211549 | 45211841 | AluJb,SINE/Alu | 6 | 1 | 85.7 |
| chr12 | 28050879 | 28051088 | MIR3,SINE/MIR | 0 | 2 | 0.0 |
| chr22 | 23351240 | 23351896 | PABL_A,LTR/ERV1 | 3 | 1 | 75.0 |
