# Supplementary material for: Methy-Pipe: An Integrated Bioinformatics Pipeline for Whole Genome Bisulfite Sequencing Data Analysis
Source: PLoS One. 2014 Jun 19;9(6):e100360. doi: 10.1371/journal.pone.0100360 (PMC4063866; doi:10.1371/journal.pone.0100360)
Supplement: Table S6 — The example list of identified DMRs. (DOCX) [file pone.0100360.s006.docx]

**Table S6.** The example list of identified DMRs

| **Chr** | **Start** | **End** | **hypo/hyper** | **Test** | | **Control** | | **Methylation density (%)** | | **P-value** | **CpG number assayed** | |
| --- | --- | --- | --- | --- | --- | --- | --- | --- | --- | --- | --- | --- |
|  |  |  |  | **Cytosine counts** | **Thymine counts** | **Cytosine counts** | **Thymine counts** | **Test** | **Control** |  | **Test** | **Control** |
| chr10 | 38816800 | 38818300 | hypo | 132 | 191 | 279 | 52 | 40.87 | 84.29 | 2.91E-08 | 30 | 25 |
| chr10 | 42383000 | 42397400 | hypo | 33862 | 28587 | 46045 | 8340 | 54.22 | 84.66 | 0.00E+00 | 719 | 601 |
| chr10 | 42596400 | 42600400 | hypo | 22042 | 18380 | 29667 | 5987 | 54.53 | 83.21 | 0.00E+00 | 193 | 178 |
| chr15 | 28834100 | 28834600 | hypo | 0 | 47 | 11 | 22 | 0 | 33.33 | 2.56E-04 | 9 | 6 |
| chr16 | 33962600 | 33963600 | hypo | 55 | 1162 | 245 | 692 | 4.52 | 26.15 | 2.78E-35 | 41 | 35 |
| chr16 | 46385500 | 46411100 | hypo | 22834 | 26581 | 31153 | 6534 | 46.21 | 82.66 | 0.00E+00 | 1195 | 1019 |
| chr17 | 36283500 | 36288000 | hypo | 824 | 441 | 1063 | 132 | 65.14 | 88.95 | 3.92E-07 | 156 | 159 |
| chr17 | 36291300 | 36292300 | hypo | 47 | 101 | 138 | 45 | 31.76 | 75.41 | 1.44E-05 | 16 | 18 |
| chr18 | 108100 | 108600 | hypo | 41 | 152 | 177 | 123 | 21.24 | 59 | 1.03E-07 | 6 | 6 |
| chr18 | 111700 | 112700 | hypo | 202 | 715 | 767 | 557 | 22.03 | 57.93 | 6.21E-28 | 59 | 60 |
| chr9 | 66455500 | 66456000 | hyper | 12 | 18 | 0 | 25 | 40 | 0 | 3.18E-03 | 5 | 5 |
