# Supplementary material for: Methy-Pipe: An Integrated Bioinformatics Pipeline for Whole Genome Bisulfite Sequencing Data Analysis
Source: PLoS One. 2014 Jun 19;9(6):e100360. doi: 10.1371/journal.pone.0100360 (PMC4063866; doi:10.1371/journal.pone.0100360)
Supplement: Table S7 — The example list of DMRs annotated with the closest genes. (DOCX) [file pone.0100360.s007.docx]

**Table S7.** The example list of DMRs annotated with the closest genes.

| **Chr** | **Start** | **End** | **hypo/hyper** | **Test** | | | **Control** | | | **Methylation density (%)** | | **P-value** | **CpG number assayed** | | **DMRs associated**  **genes** |
| --- | --- | --- | --- | --- | --- | --- | --- | --- | --- | --- | --- | --- | --- | --- | --- |
|  |  |  |  | **Cytosine counts** | **Thymine counts** | **Cytosine counts** | | **Thymine counts** | **Test** | | **Control** |  | **Test** | **Control** |  |
| chr10 | 5093000 | 5093500 | hypo | 20 | 37 | 51 | | 12 | 35.09 | | 80.95 | 8.49E-03 | 6 | 5 | AKR1C3:intron |
| chr10 | 5094500 | 5096400 | hypo | 182 | 111 | 322 | | 39 | 62.12 | | 89.2 | 2.88E-03 | 27 | 28 | AKR1C3:intron |
| chr10 | 5117500 | 5118000 | hypo | 8 | 27 | 29 | | 2 | 22.86 | | 93.55 | 1.93E-03 | 5 | 5 | AKR1C3:intron |
| chr10 | 5210900 | 5211400 | hypo | 18 | 47 | 59 | | 9 | 27.69 | | 86.76 | 2.71E-04 | 5 | 6 | AKR1CL1:intron |
| chr10 | 5237400 | 5237900 | hypo | 29 | 39 | 52 | | 3 | 42.65 | | 94.55 | 6.41E-03 | 6 | 6 | AKR1C4:promoter |
| chr10 | 5241000 | 5242000 | hypo | 62 | 94 | 221 | | 36 | 39.74 | | 85.99 | 9.31E-06 | 15 | 17 | AKR1C4:intron |
| chr10 | 5405100 | 5408700 | hypo | 280 | 408 | 640 | | 90 | 40.7 | | 87.67 | 4.22E-18 | 70 | 66 | UCN3:5UTR |
| chr10 | 5410400 | 5417300 | hypo | 596 | 640 | 1279 | | 172 | 48.22 | | 88.15 | 5.70E-22 | 126 | 125 | UCN3:5UTR |
| chr10 | 5436800 | 5437800 | hypo | 146 | 125 | 276 | | 19 | 53.87 | | 93.56 | 2.89E-05 | 19 | 19 | TUBAL3:CDS:3 |
| chr10 | 5441300 | 5441800 | hypo | 9 | 27 | 53 | | 9 | 25 | | 85.48 | 2.32E-03 | 6 | 6 | TUBAL3:intron |
| chr10 | 5486800 | 5487300 | hypo | 54 | 46 | 91 | | 3 | 54 | | 96.81 | 8.87E-03 | 7 | 5 | NET1:promoter |
